# Dietary n-3 fatty acids have suppressive effects on mucin upregulation in mice infected with *Pseudomonas aeruginosa*

**DOI:** 10.1186/1465-9921-8-39

**Published:** 2007-06-05

**Authors:** Daniel Tetaert, Maud Pierre, Dominique Demeyer, Marie-Odile Husson, Laurent Béghin, Claude Galabert, Frédéric Gottrand, Christopher Beermann, Benoit Guery, Jean-Luc Desseyn

**Affiliations:** 1 INSERM, U837, JPARC Research Centre, IMPRT, place de Verdun, Lille, France; 2 EA 3925, IMPRT, University of Lille 2 and CHRU of Lille, France; 3 CERM, Hôpital Renée Sabran, Giens, Hyères, France; 4 Numico Research, Friedrichsdorf, Germany; 5 EA 2689, IMPRT, University of Lille 2 and CHRU of Lille, France

## Abstract

**Background:**

Mucin hypersecretion and mucus plugging in the airways are characteristic features of chronic respiratory diseases like cystic fibrosis (CF) and contribute to morbidity and mortality. In CF, *Pseudomonas aeruginosa *superinfections in the lung exacerbate inflammation and alter mucus properties. There is increasing evidence that n-3 polyunsaturated fatty acids (PUFAs) exhibit anti-inflammatory properties in many inflammatory diseases while n-6 PUFA arachidonic acid (AA) favors inflammatory mediators such as eicosanoids prostaglandin E2 (PGE2) and leukotriene B4 (LTB4) that may enhance inflammatory reactions. This suggests that n-3 PUFAs may have a protective effect against mucus over-production in airway diseases. Therefore, we hypothesized that n-3 PUFAs may downregulate mucins expression.

**Methods:**

We designed an absolute real-time PCR assay to assess the effect of a 5-week diet enriched either with n-3 or n-6 PUFAs on the expression of large mucins in the lungs of mice infected by *P. aeruginosa*.

**Results:**

Dietary fatty acids did not influence mucin gene expression in healthy mice. Lung infection induced an increase of the secreted gel-forming mucin *Muc5b *and a decrease of the membrane bound mucin *Muc4*. These deregulations are modulated by dietary fatty acids with a suppressive effect of n-3 PUFAs on mucin (increase of *Muc5b *from 19-fold up to 3.6 × 10^5^-fold for the n-3 PUFAs treated group and the control groups, respectively, 4 days post-infection and decrease of *Muc4 *from 15-fold up to 3.2 × 10^4^-fold for the control and the n-3 PUFAs treated groups, respectively, 4 days post-infection).

**Conclusion:**

Our data suggest that n-3 PUFAs enriched diet represents an inexpensive strategy to prevent or treat mucin overproduction in pulmonary bacterial colonization.

## Background

*Pseudomonas aeruginosa *is an opportunistic Gram-negative pathogen known for its environmental versatility and ability to cause diseases in susceptible individuals. *P. aeruginosa *is an inhabitant of soil, water and vegetation but, unlike many environmental bacteria, can cause urinary, respiratory, and digestive tract infections usually restricted to hospitalized patients with predisposing conditions (device related infections, mechanical ventilation). Moreover, *P. aeruginosa *respiratory tract chronic infections are common in cystic fibrosis (CF) and other chronic lung conditions generating excessive inflammatory response leading to airway obstruction and structural lung damage [1].

*Pseudomonas *superinfections in CF, as well as chronic obstructive pulmonary disease (COPD), are multifactorial involving innate host immunity, pathogen characteristics and mucus properties. Chronic inflammatory diseases are mainly characterized by an inappropriate production of inflammatory mediators and chronic inflammation, which is associated with tissue damage and organ dysfunction. There is evidence that n-3 polyunsaturated fatty acids (PUFAs) exhibit anti-inflammatory properties in many inflammatory diseases while n-6 PUFA arachidonic acid (AA) favors inflammatory mediators such as eicosanoids prostaglandin E_2 _(PGE_2_) and leukotriene B_4 _(LTB_4_) that may enhance inflammatory reactions. Experimental data and clinical studies suggest that n-3 PUFAs represent potential therapeutic agents for inflammatory diseases (reviewed by Mori *and *Beilin [2]; James *et al*. [3]). For example, Shahar *et al*. reported that dietary intake of n-3 fatty acids and fish consumption play a protective effect in COPD [4]. In fact, n-3 PUFAs could improve not only inflammation but also diminish mucosal damage [5]. Concerning CF, Freedman *et al*. showed that a membrane lipid imbalance exists in both *Cftr*^-/- ^mice and in subjects with cystic fibrosis [6-8]. This imbalance may play a crucial role in the pathogenesis of CF and could be corrected by oral administration of docosahexaenoic acid (DHA).

One important factor in the morbidity and mortality of chronic airway diseases is the overproduction of mucus with altered rheologic properties (for review, see [9]), which may influence the first step of pathogen adhesion. For example, gland ducts of the airway epithelium become occluded with mucus during the first months in the CF fetus [10]. Mucins are heavily O-glycosylated proteins found in the mucus layer and responsible for its physical properties. These complex large molecules trap bacteria in the lung, and the ciliary beating draws the mucus layer up to the pharynx where normally it is swallowed (for reviews, see [11,12]). Mucins are usually subdivided into two families. The first one is made of large monomeric mucins, which are located primarily, but not exclusively, at the cell surface. The other one is made of the five, large secreted gel-forming mucins *MUC6*, *MUC2*, *MUC5AC*, *MUC5B *and *MUC19*, which are conserved between human and mouse [13-15] and form oligomeric structures [9,16,17]. Among the identified mucins, the large anchored-mucin *MUC4 *and the two large gel-forming mucins *MUC5B *and *MUC5AC *seem highly expressed in normal and/or abnormal human lung while *MUC2 *is almost not detectable [18]. At the cellular level, *MUC5AC *is expressed in the goblet cells of main bronchi and bronchioles and *MUC5B *is expressed in bronchiolar epithelium and in submucosal gland epithelium while MUC4 transcripts are detected in the epithelium of main bronchi and bronchioles [19-21]. While many studies have focused on gene regulation of both *MUC2 *and *MUC5AC *using mainly airway cell cultures and explants, regulation of *MUC4 *and *MUC5B *in airway diseases have been ignored to date.

In this study, we hypothesized that dietary intakes of n-3 PUFAs decrease mucin production in chronic airway diseases that likely decreases the mucus visco-elasticity and, on the other hand, that a diet enriched in n-6 PUFAs exacerbated mucin expression. We determined the mucin expression pattern in lung of healthy wild-type mice and then *in vivo *mucin regulation of *Pseudomonas*-infected mice that were fed for 5 weeks with a diet enriched in either n-3 PUFAs or n-6 PUFAs compared to a control rodent diet with equal fat content.

## Methods

### Mice

Five-week-old male C57BL/6 mice were purchased from Charles River (France) and housed five per cage in filter-capped cages with hardwood bedding. Cages are placed into a ventilated cabinet in a quiet room at the University of Lille on a 12:12 hour light/dark cycle and were kept at a constant room temperature (24°C). Animals were allowed to acclimatize for at least one week prior the start of the experimental procedure. They were randomized to be fed for 5 weeks with either a control diet (C), a diet supplemented with n-3 or n-6 PUFAs. Tap water and sterilized food were continuously available throughout the study. The three formulations used are identical to the AIN-93G [22] except that the soybean oil has been replaced by three isocaloric and iso-fat content mixtures (Table 1). Diet 1 is dominated by EPA and contains also lower amounts of other n-3 PUFAs (ALA, STA, DHA) while diet 2 is dominated by arachidonic acid (AA) (n-6 PUFAs). This study is a subset of the mice used in a recent publication [23]. All procedures were in accordance with the French Guide for the Care and Use of Laboratory Animals and with the guidelines of the European Union.

**Table 1:** Composition in fatty acids of the three diets (mg/100 mg of FA)

Group		ω3	ω6	control
Fatty acid				
S-FA		11.7	25.9	31.3
MUFA		52.0	42.0	49.9
ALA	18:3n-3	**3.7**	3.1	3.2
STA	18:4n-3	**0.8**	< 0.1	< 0.1
EPA	20:5n-3	12.7	< 0.1	< 0.1
DHA	22:6n-3	**3.9**	0.1	< 0.1
LA	18:2n-6	14.2	12.8	14.5
GLA	18:3n-6	< 0.1	1.0	< 0.1
AA	20:4n-6	0.7	**13.5**	0.1

abbreviations : FA, fatty acids; S-FA, total saturated FA; MUFA, mono-unsaturated FA; ALA, alpha-linolenic acid; STA, stearidonic acid; EPA, eicosapentaenoic acid; DHA, docosahexaenoic acid; LA, linoleic acid; GLA, gamma-linolenic acid; AA, arachidonic acid

### Quantitative real-time PCR

**Figure 1:**
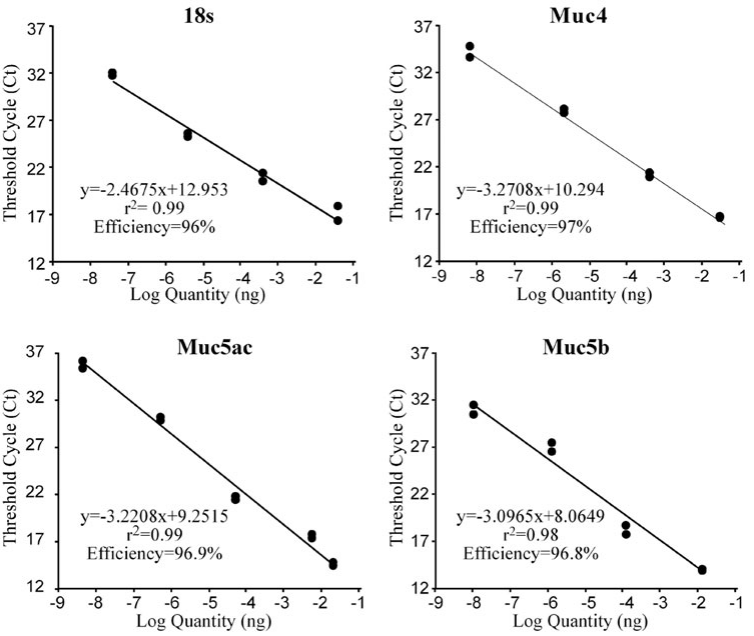
Graphical representation of linear regression analyses to validate the quantitative real-time PCR (qPCR) assay. Dilutions of plasmid cDNA were amplified by qPCR in duplicate reactions, and standard curves were created by plotting the cycle threshold (C_t_) values versus DNA quantity (Log quantity) in ng for *18s*, *Muc4*, *Muc5ac *and *Muc5b*.

Primer and TaqMan probe sequences were selected using the Primer3 Output program Technology [24] from the Massachusetts Institute of Technology. Primers and probes (Table 2) have been chosen within the 3'-end of mouse Muc cDNAs. To avoid amplification of contaminating genomic DNA, primers and/or the probes have been designed on different exons or overlap exon-exon junctions. To quantify mucin expression obtained by real-time PCR, we used the standard curve method. The 18s rRNA was chosen as an internal positive control. For each mucin studied and for the *18s *gene, reverse-transcribed RNA extracted from mouse lung was amplified (see below) and amplification products were cloned into the 3957-bp pCR4-TOPO vector (Invitrogen, France). Isolation of plasmid DNA was carried out with the QIAprep Spin Miniprep Kit (Qiagen). Positive clones were reconfirmed by digestion with the *Eco*RI restriction enzyme and inserts sequenced on both strands on Licor 4000 using universal oligonucleotides. Serial dilutions of each Qiagen-purified plasmid with the cDNA insert were prepared and DNA concentrations measured using the spectrofluorometer TD-360 (Fisher-Bioblock, France) and the Hoechst 33258 dye. Serial dilutions of plasmid cDNAs were amplified by qPCR in duplicate reactions, and standard curves were created by plotting the log of the input plasmid cDNA quantity (ng) versus cycle threshold (C_t_) values for *Muc4*, *Muc5ac*, *Muc5b *and the *18s *(Figure 1) allowing to determine the copy number for each sample.

### Preparation of the bacterial inoculum

The methodology was adapted from Cash *et al*. [25]. Briefly, *P. aeruginosa *(PAO1 strain) was incubated in 125 ml of tryptic soy broth at 37°C in a rotating shaking water bath for 8 hours. The culture was then washed twice, and resuspended in phosphate-buffered saline. The resulting bacterial suspension was 1 × 10^9 ^CFU/ml. A sample of 1 mL of this suspension was mixed in agarose and mineral oil (Sigma Diagnoses, St Louis, USA) at 56°C. The resulting oil-agarose emulsion was cooled to obtain agar beads. Dilutions of the final suspension were cultured to determine the CFU of the final inoculum. Thirty mice (10 per group) were inoculated per batch of agarose beads.

**Table 2:** Primer and probe sequences for murine mucins

Mucin		Sequence (5'-3')	Product length
Muc4	FW	GTCCACTTCTTCCCCATCTC	
	RV	GTAGCCTTTGTAGCCATCACAT	
	PB	CCAGGACCAGATGGCTCTGAACCT	102 bp
Muc5b	FW	GCACGTAAATGCGACTGTCT	
	RV	ATGGACCTTGCTCTCCTGAC	
	PB	TATCCAAGTACTCCATGGAGGCCC	133 bp
Muc5ac	FW	TCCCTTACCTAACCAGCAGAA	
	RV	GGGAGTACATGGAGATGCTGT	
	PB	GAGGGCCCAGTGAGCATCTCCTACT	118 bp

FW, forward primer; RV, reverse primer; PB, TaqMan probe dual-labeled with 5'FAM and 3'TAMRA.

### Experimental infection

Mice were anesthetized with sevoflurane (Sevorane™, Abbott, UK), and placed in dorsal recumbency. Transtracheal insertion of a 24-G animal feeding needle was used to instill 60 μL of a 1:4 dilution of the agarose beads (2 × 10^5 ^CFU/mouse). The mice were then allowed to recover. The inoculation of mice is a critical step [26] and for a better reliability a single operator conducted it. After instillation, mice were observed daily for clinical signs, such as posture and ambulation. Two experimentally infected mice were killed before the end of the experiment due to their poor ambulation and visual sign of painful posture. A few other infected mice died during the night and their carcass eliminated whereas no mice inoculated with sterile beads died.

### Quantitative bacteriology

All mice were sacrificed using carbon dioxide followed by cervical dislocation. The lungs were excised aseptically on the first, the fourth or the seventh day after the inoculation or just after 5 weeks of diet (control mice, day 0). These lungs were then homogenized in PBS buffer, and the samples were cultured quantitatively by serial dilution on BCP agar plates (Biomerieux laboratories, Lyon, France). The plates were incubated at 37°C and *P. aeruginosa *colonies were counted after 24 h.

### Expression of Muc genes

Uninfected and PAO1 instilled mice were sacrificed after 1, 4, or 7 days post- infection by cervical dislocation. The lungs were collected, washed into sterile PBS, rapidly frozen in liquid nitrogen and stored at -80°C until RNA extraction. Total RNA was extracted using TriReagent (Molecular Research Center, Inc., Euromedex, France) following manufacturer's protocol followed by precipitation in ethanol and resuspension in ribonuclease-free water. Single-stranded cDNA was generated from 2 μg of total RNA using the First -Strand cDNA Synthesis Kit (Clontech, Ozyme, France) and random hexamers according to the manufacturer's instructions. The 20 μL of cDNA was diluted with 80 μL of DEPC-water. Duplex PCR amplification was carried out in 25-μL reaction volumes containing 2 μL of the first strand cDNA, 10 pmol of each primer for a given mucin studied, 5 pmol of each mucin probe and 2× TaqMan Universal PCR Master Mix (Applied Biosystems) containing 1 pmol of the 18s-primers (sense and antisense), 5 pmol of the 18s-probe (5' Vic reporter dye), 5,6-carboxy-x-rhodamine (ROX), the *Taq *DNA polymerase and the requisite buffers. Probes (Applied Biosystems, France) were labeled with 6-carboxy-fluorescein phosphoramidite (FAM) at the 5' end and as quencher 6-carboxy-tetramethyl-rhodamine (TAMRA) at the 3' end. The fluorescence intensity of the reporter label was normalized to the rhodamine derivative ROX as a passive reference label present in the buffer solution. The system generated a kinetic amplification plot based upon the normalized fluorescence. Amplifications were performed on the ABI Prism 7700 Sequence Detection System (Applied Biosystems). Quantitative analysis was conducted using an ABI7700 Real-Time Detection System. The PCR reaction ran for 45 cycles of a two-step PCR amplification (95°C for 10 minutes and 60°C for 1 minute). All reactions were run in triplicate. Negative controls were carried out with water instead of cDNA.

### Data and statistical analysis

**Figure 2:**
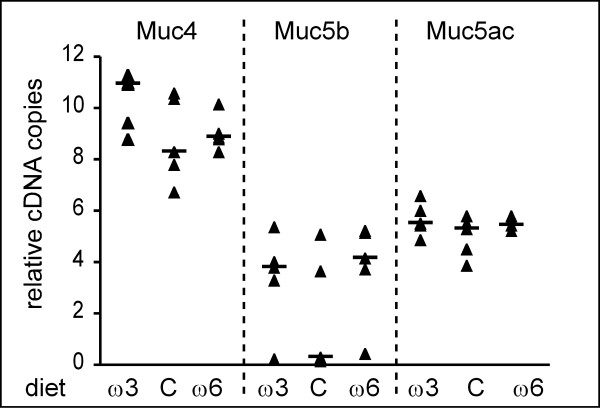
Gene expression analysis in lung by absolute qPCR of mouse *Muc4*, *Muc5ac *and *Muc5b *transcripts normalized with the *18s*. Five healthy mice fed with the control diet for 5 weeks were used in each group. Ratios between the absolute amounts of the three Muc copy numbers and the *18s *copy number is given in Log_10 _(Muc/18s)+11 due to the high difference between the *Muc4 *level expression and the two other Mucs gene expression levels.

**Figure 3:**
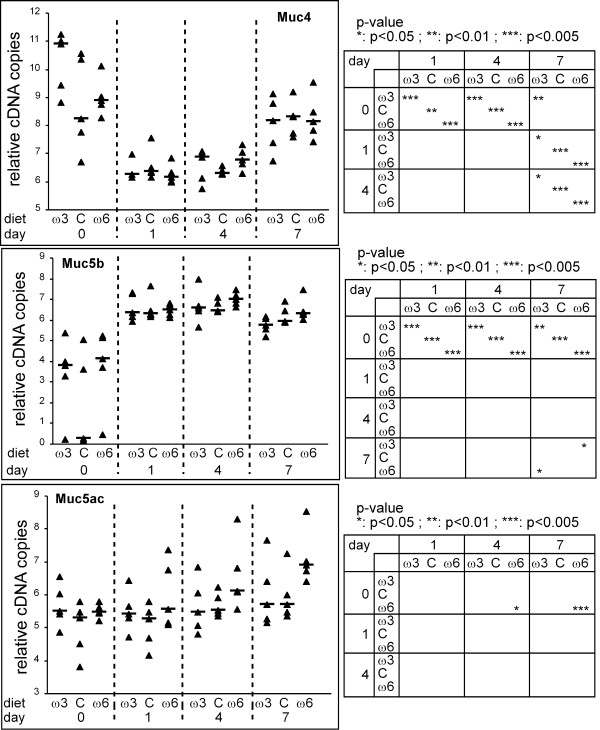
Effect of the diet on the mouse *Muc4*, *Muc5b *and *Muc5ac *gene expressions of mice inoculated with sterile agarose beads. Values represent the Log_10 _copy number of each Muc transcript normalized with the *18s *(log(Muc/18s))+11. Exact P-values were calculated to compare the effect between days for a given diet (ω3: n-3 PUFA treated group; C: control diet group; ω6: n-6 PUFA treated group) or between diets for a given day. Significant P-values are given in 2 × 2 tables.

**Figure 4:**
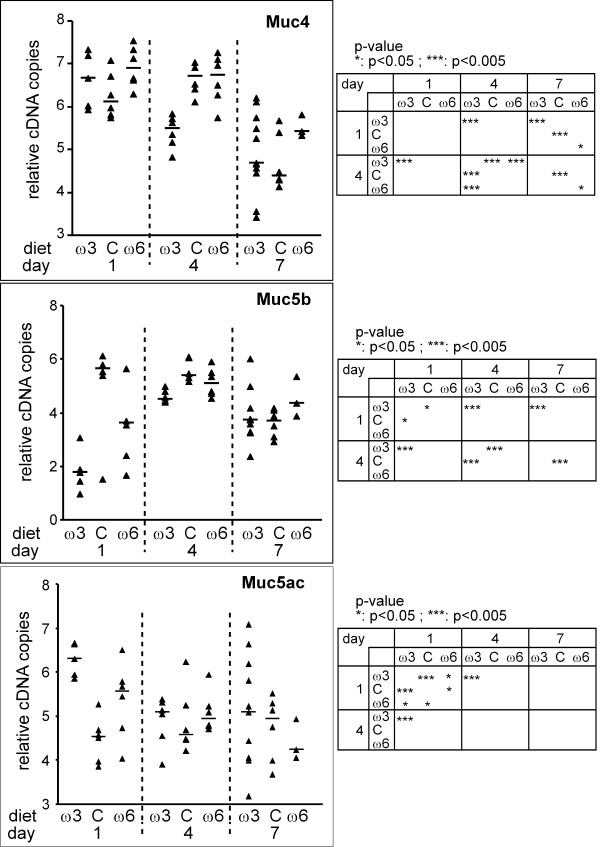
Suppressive effect of an n-3 PUFAs enriched diet on the Muc gene expression of mice infected with *P. aeruginosa*. Values represent 6+log [(Muc of infected lung/18s of infected lung)/median(Muc of sterile beads/18s of sterile beads)]. Exact P-values were calculated to compare the effect between days for a given diet (ω3: n-3 PUFA treated group; C: control diet group; ω6: n-6 PUFA treated group) or between diets for a given day. Significant P-values are given in 2 × 2 tables.

cDNA copies of Muc transcripts were adjusted with the number of cDNA copies for the *18s *and Log transformed values were represented (Figures 2, 3, 4). For PAO1-inoculated experiment (Figure 4), each measured value has been divided by the median of the counterpart group of mice inoculated with sterile agarose beads. Comparisons were analyzed using the statistical package StatXact^® ^6.0 (Cytel Studio) for exact nonparametric inference. Wilcoxon-Mann-Whitney one-sided p-values were calculated to compare differences between groups (between diets for each day and between days for each diet) and given in Figures 3, 4. P values are given in 2 × 2 tables on figures 3 and 4. Correlations between variables were calculated by using Spearman's rank correlation tests (*r*_s_) with the Monte Carlo option. A p-value ≤ 0.05 was considered statistically significant.

## Results

Mice were fed for 5 weeks prior *P. aeruginosa *(PAO1) infection with either a control diet (C) or a diet enriched with n-3 PUFAs or n-6 PUFAs (Table 1). This model mimics chronic lung infection in mice [25-28] as they lost weight after infection and bacteria could still be recovered at the termination of the study [23]. Bacterial clearance was analyzed over the 7 days of the experiment. The number of bacteria dropped over time in each group, and a comparable pattern was observed for each diet with no difference between the three groups [23].

To study mucin gene expression, we designed a fluorescence based PCR assay to determine an exact and absolute quantification of the mouse *Muc5b*, *Muc5ac *and *Muc4 *RNAs allowing comparisons of expression levels between the three different Muc genes. The housekeeping gene *18s *was used as an internal positive control and to normalize each Muc gene expression level. The cDNA amplification products of mouse *Muc4 *(102 bp ; table 2), *Muc5b *(133 bp), *Muc5ac *(118 bp) and mouse *18s *(187 bp) were cloned into the pCR4 plasmid vector. Serial dilutions of these cDNA plasmids were used to establish standard curves, and cycle threshold (C_t_) values were determined to be between 8 and 13 (Figure 1). C_t _values represent the number of amplification cycles required to produce a specific amount of fluorescence during the exponential phase of the quantitative PCR. Consequently, the higher the C_t _value, the lower the concentration of the template being amplified. C_t _values increased linearly with decreasing input cDNA plasmids. The average *r*^2 ^values for the 18s and the Muc genes assayed were greater than 0.98 (Figure 1). Efficiency of our Muc PCR assays varied (mean slope) from -3.10 to -3.27 while the efficiency of the commercially *18s *was -2.47.

We performed qPCR on 15 mice that were non-inoculated (day 0, 5 mice/group), 45 inoculated with sterile beads and sacrificed at days 1, 4 and 7 (5 mice/group/day) and on infected lung tissues from mice of the n-3 PUFAs, control and n-6 PUFAs diet groups that were killed at days 1 (5, 6 and 6 mice, respectively), 4 (6 mice/group) or 7 (11, 6 and 3 mice, respectively). Figure 2 shows the relative copy numbers of *Muc4*, *Muc5b *and *Muc5ac *normalized with the *18s *for mice fed with the three diets. The expression level of *Muc4 *in lung of healthy mice was higher than the expression levels of both *Muc5b *and *Muc5ac *RNAs. Medians of normalized Muc genes for control groups were 1.8 × 10^-3^, 2 × 10^-11 ^and 2 × 10^-6 ^for *Muc4*, *Muc5b *and *Muc5ac*, respectively, pointing out the high level of *Muc4 *in comparison with the two secreted gel-forming mucins. We did not observe any significant impact of the diet on the regulation of the three Muc genes studied.

Inoculation with sterile agarose beads induced a significant downregulation of *Muc4 *and an upregulation of *Muc5b *(Figure 3). At day 7, the level of *Muc4 *increased towards its baseline level (day 0). For each day, the diet has no impact on the two mucins gene expression except at day 7 on the *Muc5b *transcripts where the level of expression is n-3 < n-6 (p < 0.05). Concerning the *Muc5ac *gene, levels of the RNA transcript between the groups were similar over time with the exception for the n-6 PUFAs treated group between days 0/4 and 0/7 where the AA-enriched diet played an enhanced effect. In conclusion, the diet has almost no effect on mucin gene expression of mice inoculated with sterile agarose beads.

We next examined whether diet had an impact on *Pseudomonas*-infected mice. For each group at each day, the Log10 transformed value measured was normalized with the mucin median value of the corresponding group where mice were inoculated with sterile agarose beads. Membrane-bound *Muc4 *expression decreased over time (Figure 4). On the fourth day after infection, the n-3 PUFAs enriched diet has a high suppressive effect on the level expression of *Muc4 *(p < 0.005). *Muc5b *expression increased significantly for the n-3 PUFAs treated group between days 1 and 4. The expression levels tend to go down at day 7 with a statistically decrease for the control diet group. At days 1 and 4, the peak of expression of the large secreted gel-forming mucin *Muc5b *was higher for the control group diet in comparison with the n-3 PUFAS treated group suggesting a suppressive effect of n-3 PUFAs on inoculated mice. *P. aeruginosa *lung infection did not appear to impact *Muc5ac *expression over time, regardless of diet. Level of expression is higher at day 1 for the n-3 PUFAs treated group comparing to the two other groups. This peak is followed by a decrease at day 4. Additionally, we noticed a large dispersion of samples of the n-3 PUFAs treated group for the three Mucs studied at day 7.

Associations between the three-studied Muc gene expressions were assessed using Spearman's s rank correlation coefficient. There was no statistically significant association between *Muc4 *and either *Muc5ac *or *Muc5b *at day 0 and at day 1. However, Spearman's rank correlation indicated a strong positive correlation at day 4 (r_s _= 0.67 ; p < 0.004 and r_s _= 0.84 ; p < 0.0001), for *Muc4*/*5ac *and *Muc4*/*5b*, respectively and at day 7 (r_s _= 0.9 ; p < 0.0001 and r_s _= 0.76 ; p < 0.0001) for *Muc4*/*5ac *and *Muc4*/*5b*, respectively. Concerning *Muc5ac *and *Muc5b*, the two gene expressions were statistically correlated at days 1 and 7 (r_s _= -0.57 ; p < 0.03 and r_s _= 0.81 ; p < 0.0001, respectively). Spearman's rank-order correlation coefficient did not show any relationships between the bacteria number and the three Muc genes except for *Muc4 *at day 7 (r_s _= -0.48 ; p < 0.04).

## Discussion

Mucus hypersecretion, viscoelastic alteration and mucin overproduction are the hallmarks of many lung diseases such as CF [9,17,29] where *P. aeruginosa *is the principal pathogenic microorganism. The properties of the mucus layer are dictated by the water content and the mucin layer composition. *MUC5AC*, *MUC5B *and *MUC4*, which are conserved mucins between human and rodent [30,31], are three predominant large mucins expressed in the lung. Many studies have focused on *MUC5AC *and *MUC2 *regulation in airway diseases using mainly airway cell and explant cultures [32-34] while the expression of the oligomeric mucin *MUC5B *and the membrane-bound mucin *MUC4 *have not yet been investigated. However, biochemical studies on human samples have shown that the aberrant physical properties of the mucus gel obstructing the airways from an individual that died in *status asthmaticus *are due to *MUC5B *and not *MUC5AC *[35,36]. Many, if not all, investigations that used human secretions encountered biased results due to the false status of the patient studied, non-controlled environmental parameters or difficulties to analyze *in vivo *mucins production pointing out the benefit of animal models in CF studies. Using a precise and reliable assay we (i) determined here the relative expression of lung mucins of healthy mice, mice inoculated with sterile agarose beads and mice infected with *P. aeruginosa *and (ii) examined the effects of a diet enriched either with n-3 PUFAs or n-6 PUFAs on mucin expression following experimental mouse lung inoculation.

The model of infection we used does not mimic perfectly a chronic lung infection, as the number of bacteria declined over time and several lung mice that were inoculated with PA01 were culture-negative at day 7. However, this model is still the best one available in mice [27,28]. A single operator trained for delivering a uniformity of bead preparations from experiment to experiment and for inoculating mice performed these two steps in order to minimize caveats associated with this model (e.g., variability inherent to operator for the instillation procedure, in making agarose beads [26]).

To follow the mouse Muc gene expression that likely reflects protein synthesis [5,37,38], we designed an absolute PCR quantification assay instead of the often-used comparative C_t _method. One advantage of the absolute qPCR is that the efficiency of the housekeeping gene used as an internal reference and the target Muc genes must not be necessary identical. Furthermore, absolute qPCR allows a more precise and sensitive RNA quantification with reliability between different laboratories. In our mouse model, we followed *Muc5b*, *Muc5ac *and *Muc4 *while the *Muc2 *messenger was barely undetectable in lung tissue and did not show any variation prior or after instillation by sterile or PAO1-laden agarose beads (data not shown). This study showed for the first time that diet has an impact and could modulate lung mucin gene expression in an *in vivo *model of bacterial infection.

As it is well-known, rodent models only partially resemble the native architecture and abundance of submucosal glands in the human airways [39], it is therefore not surprising that *Muc5b *is almost undetectable using qPCR in the lungs of healthy mice. In human, it has been reported that *MUC5AC *expression, measured using real-time RT-PCR, is much higher than both *MUC5B *and *MUC4 *in homogenates of endobronchial biopsies from healthy subjects or subjects with asthma [40]. Here we showed in mouse lung that *Muc4 *is highly expressed in comparison to *Muc5b *and *Muc5ac*. This could be due to the species studied and/or the "healthy" status of the human biopsies used by others and/or the RT-PCR quantification strategy. Moreover, we chose an absolute quantification method using primers directed at the 3'-end of the Muc genes with oligonucleotides belonging to different exons while in the previous work authors quantified *MUC4 *expression using a relative qPCR strategy with oligonucleotides chosen within exon 2 of the gene.

Yanagihara *et al*. showed that intratracheal injection of 100 μg of *P. aeruginosa *lipopolysaccharide (LPS) induced mucus cell metaplasia. Northern blot analysis using 3 mice showed an increase of the *Muc5ac *messenger with a peak at day 4 followed by a decrease of *Muc5ac *RNA [41]. In this present study, we did not observe major modification in the *Muc5ac *expression with the exception of an upregulation at day 7 for the n-6 PUFAs treated group inoculated with sterile beads (Figure 3).

PAO1 lung inoculation induced two opposite expression patterns of *Muc5b *and *Muc4*: *Muc5b *is highly upregulated with a peak at day 4 showing a suppressive effect of n-3 PUFAs on the *Muc5b *response to the infection while *Muc4 *expression decreased to the lowest level at days 1 or 4 for sterile beads and at day 7 for *Pseudomonas*-laden agarose beads. In parallel with the bacterial clearance by ciliary transport, expression of *Muc5b *decreased almost to baseline levels on day 7. The increase of *Muc5b *messenger may reflect either a transdifferentiation of ciliated cells to a secretory phenotype or differentiation of hypothetical progenitor cells [38].

It is now well known that consumption of fish oil containing n-3 PUFAs reduces the risk of many human diseases [42]. Dietary lipid manipulation may affect different immune parameters and the immune modulation induced by dietary fatty acids may be applied by the improvement of inflammatory disorders. A dietary immune regulation with a defined blend of specific long-chain polyunsaturated fatty acid species should represent an interesting alternative to immune suppressive molecules, like corticosteroids, which often causes side-effects. Compared to other studies that used fish oil supplementation or uncontrolled vegetable oil extractions, we used two fat isocaloric and isofat content blends. One is dominated by eicosapentanoic acid (EPA) -but contained other n-3 PUFAs- that act as a competitive inhibitor of AA conversion to the pro-inflammatory key mediators PGE_2 _and LTB_4 _while the other diet is dominated by AA, which gives rise to PGE_2 _and LTB_4 _(reviewed by James *et al*. [3]). On the other hand, PUFAs are also candidates to directly influence gene expression that is connected to diseases and then to enhance epithelial protection during inflammation and/or pathogenic bacterial colonization. PUFAs integrated into cellular membranes could also influence cell function and membrane integrity as DHA and AA compete for the same elongation and desaturation enzymes and for the site of esterification of phospholipids. The switching of AA by DHA leads to a change in regulatory fluidity and membrane trafficking [43,44].

The present study shows that dietary PUFAs did not influence mucin gene expression in the lungs of healthy mice after 5 weeks of diet (control mice and mice inoculated with sterile beads). As the three experimental diets only differed in their content of PUFAs, differences in fatty acids are most likely responsible for the regulation of lung mRNA transcripts of mucins after infection by *P. aeruginosa *in agreement with Spearman's rank correlation. The regulation is modulated by the PUFAs intake with a suppressive effect of n-3 PUFAs on mucin upregulation and/or possibly an enhanced effect on the same Muc genes by n-6 PUFAs. This work is a part of a larger study and it should be noted that mice exposed to high n-3 PUFAs intakes and infected by *P. aeruginosa *exhibited a lower mortality than mice from the control group and mice fed with the diet rich in n-6 PUFAs [23] at day 4 after infection while survival rate of mice inoculated with sterile agarose beads was 100% at day 7 regardless of diet. One limit of our study is that a few mice inoculated with PAO1 died before day 4 (more in the control group and n-6 treated PUFAS group compared to n-3 PUFAs treated group) and were therefore not studied rending interpretations difficult for day 1.

Taken together, these data suggest that dietary intake of n-3 PUFAs reduces the lung damage by *P. aeruginosa*. Nieto *et al*. draw a similar conclusion about n-3 PUFAs and intestinal damages in rats with experimental ulcerative colitis where n-6 PUFAs enhanced tissue responsiveness to cytokines and n-3 PUFAs had opposite effects [5]. This suggests that overproduction of large mucins that likely leads to airway mucus obstruction in *Pseudomonas aeruginosa *chronic pneumonia may be counterbalanced by a n-3 PUFAs enriched diet. Further studies will be needed to confirm the relationship between mucin expression levels and mucus obstruction and to determine if the beneficial suppressive effects of dietary n-3 PUFAs on mucin expression used a direct or pro-inflammatory cytokine-dependent mechanism.

## Conclusion

In summary, lung infection by *P. aeruginosa *induced a disregulation of the large mucins *Muc5b *and *Muc4*. These modifications of mucin expression are modulated by dietary long-chain fatty acids with a beneficial suppressive effect of n-3 PUFAs confirming our first hypothesis. This strongly supports that malnutrition can compromise pulmonary defenses against *P. aeruginosa *colonization [45].

## Abbreviations

CF, cystic fibrosis; COPD, chronic obstructive pulmonary disease; C_t_, cycle threshold; FA, fatty acids; S-FA, total saturated FA; MUFA, mono-unsaturated FA; PUFAs, polyunsaturated fatty acids; ALA, alpha-linolenic acid; STA, stearidonic acid; EPA, eicosapentaenoic acid; DHA, docosahexaenoic acid; LA, linoleic acid; GLA, gamma-linolenic acid; AA, arachidonic acid ; LPS, lipopolysaccharide.

## Competing interests

The author(s) declare that they have no competing interests.

## Authors' contributions

DT carried out the molecular genetic studies and helped to draft the manuscript. MP and MOH were involved in acquisition and interpretation of bacterial data. DD performed the nucleotide sequencing, RNA extraction and helped in performing qPCR. LB assisted in experimental design and statistic analyses. CG intellectually supported the research, and critically revised the manuscript. FG and CB supervised the research and analysis and critically revised the manuscript. BG participated in the design of the study, in the animal instillation and revised the draft. JLD conceived the study, participated in its design and in molecular biology experiments and drafted the manuscript.

All authors read and approved the final manuscript.
